# The role of pyroptosis in endothelial dysfunction induced by diseases

**DOI:** 10.3389/fimmu.2022.1093985

**Published:** 2023-01-09

**Authors:** Jin Ju, Yanyan Liu, Haihai Liang, Baofeng Yang

**Affiliations:** ^1^ Guangdong Key Laboratory for Biomedical Measurements and Ultrasound Imaging, National-Regional Key Technology Engineering Laboratory for Medical Ultrasound, School of Biomedical Engineering, Shenzhen University Medical School, Shenzhen, China; ^2^ Guangdong Provincial Key Laboratory of Tumor Interventional Diagnosis and Treatment, Zhuhai People’s Hospital, Zhuhai Hospital Affiliated with Jinan University, Jinan University, Zhuhai, Guangdong, China; ^3^ Key Laboratory of Cardiovascular Research, State-Province Key Laboratories of Biomedicine-Pharmaceutics of China, Ministry of Education, Department of Pharmacology, College of Pharmacy, Harbin Medical University, Harbin, Heilongjiang, China; ^4^ Research Unit of Noninfectious Chronic Diseases in Frigid Zone (2019RU070), Chinese Academy of Medical Sciences, Harbin, Heilongjiang, China

**Keywords:** pyroptosis, endothelial dysfuction, organ injury, NLRP3, caspase1, cell signaling, drug treatment, protein protein interaction

## Abstract

Most organs in the body rely on blood flow, and vesicular damage is the leading cause of injury in multiple organs. The endothelium, as the barriers of vessels, play a critical role in ensuring vascular homeostasis and angiogenesis. The rapid development of risk factors in endothelial injuries has been seen in the past decade, such as smoking, infectious, and diabetes mellites. Pyroptotic endothelium is an inflammatory mode of governed endothelial cell death that depend on the metabolic disorder and severe infectious such as atherosclerosis, and sepsis-related acute lung injury, respectively. Pyroptotic endothelial cells need GSDMD cleaved into N- and C-terminal by caspase1, and the cytokines are released by a pore constructed by the N-terminal of GSDMD in the membrane of ECs, finally resulting in severe inflammation and pyroptotic cell death. This review will focus on the patho-physiological and pharmacological pathways of pyroptotic endothelial metabolism in diseases. Overall, this review indicates that pyroptosis is a significant risk factor in diseases and a potential drug target in related diseases.

## 1 Introduction

Pathological conditions could cause cell death, and cells could actively participate in the process of cell death (1). These regulated cell death (RCD) modes have contributed to the influence of human patho-physiological conditions such as embryonic development, homeostatic maintenance, and disease pathology (2). Adult organs are made up of more than thirty trillion cells, and millions of cells are vanished by programmed cell death (PCD) daily and replaced by freshly same cells to ensure the functions of organs. PCD is a kind of cell death due to incidents in cells, such as apoptosis (3). But dissimilar to apoptosis, the latest finding of RCD, pyroptosis, displays a preliminary disturbance of the integrity of the plasma membrane, leading to extracellular spillage of intracellular contents (4). Epigenetic modifications, such as carbon 5 methylation and m^6^A methylation, are involved in the onset and progression of cell death (5, 6). dysregulation of the epigenome drives aberrant transcriptional programmes that promote multitude of different diseases, including cancer, chronic pulmonary disease and obesity (7, 8).

The endothelium, as the barrier of the vascular arterial, venous, and lymphatic vessels, is vital for multiorgan health. Endothelial cells (ECs), a continuous cells monolayer lining the blood vessel wall, are a significant element of maintaining vascular homeostasis, anti-inflammatory, and antithrombosis (9). Endothelial cells are differentiated from endothelial progenitor cells and regulated by epigenetics (10, 11). Vascular endothelium dysfunction could induce multiple phenomena such as vasospasm, increasing oxidation stress, inflammation, leukocyte, macrophage adhesion, and so on (12). There are studies that many fatal diseases are related to endothelium dysfunction, like atherosclerosis, sepsis, diabetes mellitus, and stroke.

In the last few years, experimental and clinical research has displayed a new clue on endothelium dysfunctions. This paper aims to review related articles that have progressed in this field.

## 2 Functions of endothelial cells

Blood vessels have various functions in different organs. For example, blood vessels provide organs with oxygen and nourishment, and lymphatic vessels absorb and filter tissue fluid from organs (13, 14). Although blood vessels mostly remain inert for the whole of adulthood, they can also form new vessels rapidly when injured or in pathological situations. The ECs of the blood vessel lining play a crucial role in the development of a vessel. As a critical aspect of neovascularization, capillary sprouting is established by interactions among three EC subtypes, such as tip cells, stalk cells, and quiescent phalanx cells, and every type of ECs has a special role in this process (15).

The endothelium, the largest organ and may be one of the most various organs in the body, play multiple physiological roles, including the provision of a non-thrombogenic, nonadherent, and permeability surface, formation and secretion of molecules and cytokines such as nitric oxide (NO), maintenance of the basement membrane collagen and proteoglycans upon which they rest (9). ECs in different places (arterial, microvascular, venous, etc.) show multiple functions in multiple conditions; thus, their activated mechanisms are different (16). For example, pulmonary microvascular ECs are more sensitive to oxidative phosphorylation and ATP levels than arterial ECs (17). Brain ECs have more mitochondria and are more dependent on oxidative metabolism than peripheral blood vessels (18). Pathological conditions are also associated with EC heterogeneity and metabolic consequences. Growing evidence indicates that pyroptosis can work as an effective defense against pathological conditions, but excessive endothelial pyroptosis is the pathogenesis in many diseases, such as stroke, atherosclerosis, and acute lung injury.

## 3 Pyroptosis

Pyroptosis is a proinflammatory mode of RCD that depends on the synthetic action of inflammatory proteases named cysteine-dependent aspartate-specific protease (caspase) (19). Pyroptosis was first called (‘pyro’ means fire or fever, ‘ptosis’ means denote a falling) to imply a fire-like inflammatory of this mode RCD in 2001 (20). Interestingly, pyroptotic cells undergo early plasma membrane permeabilization, like necroptosis and accidental necrosis. The process of pyroptosis nonetheless shares some characteristics with apoptosis, even though it does not result in cell death and is generally considered immunologically silent (21). Pyroptosis is crucial to protect against foreign microbe, however, it can also lead to damage while unrestrained. It is reported excessive inflammasome promote multiple acute diseased (including sepsis, disseminated intravascular coagulation, cytokine release syndrome etc.) and chronic diseases (including atherosclerosis, diabetes, ischemic stroke etc.) (22).

**Figure 1:**
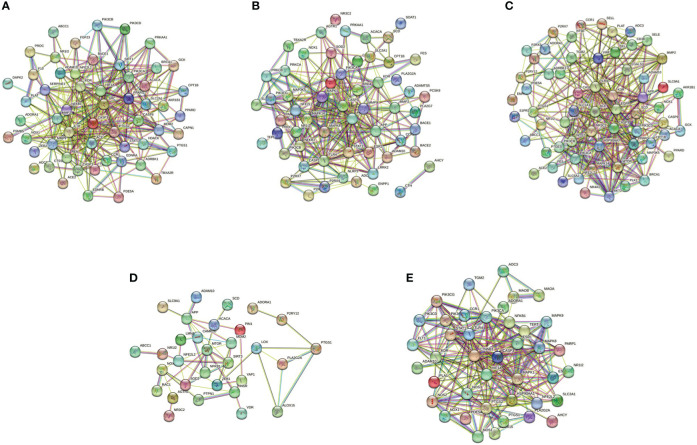
PPI network of five compounds targets in endothelial dysfunction diseases. **(A)**, Z-VAD-FMK; **(B)**, MCC950; **(C)**, VX-765; **(D)**, Disulfiram; **(E)**, Dimethyl fumarate. Database of SEA (https://sea.bkslab.org), Super-PRED (https://prediction.charite.de), PharmMapper (http://www.lilab-ecust.cn/pharmmapper/), and SwissTargetPrediction (http://www.swisstargetprediction.ch) was used to predict therapeutic targets of compounds and used a database of DisGeNET (https://www.disgenet.org/) to predict the pathogenic target of endothelial dysfunction in diseases. Then shared regulatory network was determined using Venny2.1 (https://bioinfogp.cnb.csic.es/tools/venny/) and made the PPI network by the STRING database (https://string-db.org/).

**Figure 2:**
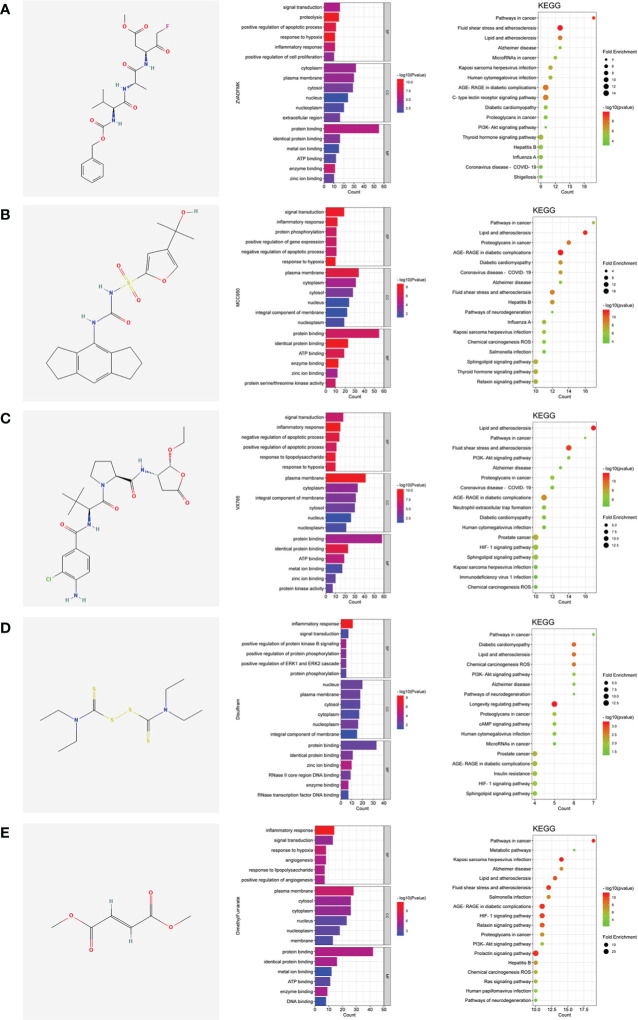
The shared regulatory targets of the GO and KEGG enrichment analyses. **(A)**, Z-VAD-FMK; **(B)**, MCC950; **(C)**, VX-765; **(D)**, Disulfiram; **(E)**, Dimethyl fumarate. The analyses of GO and KEGG were carried out by DAVID Knowledgebase (https://david.ncifcrf.gov). The images were made by bioinformatics (http://www.bioinformatics.com.cn).

The pyroptotic pathway is an important target for drugs because it plays a pivotal role in various diseases, such as infectious, stroke, sepsis, and diabetes mellites (21, 23, 24). Currently, several compounds, such as VX-765, are being tested for the treatment of pyroptosis-related diseases (25–27). The STRING database made those compounds a protein-protein interaction (PPI) network in this review (**Figure 1**), and the Gene Ontology (GO) and Kyoto Encyclopedia of Genes and Genomes (KEGG) enrichment analyses demonstrated those compounds might relieve endothelial dysfunction in atherosclerosis or infections respond to inflammation (**Figure 2**) (28–34). Some danger signals (e.g., cytokines, etc.) are released while cells have died; perhaps some are even released in primary pyroptosis (35). These signaling molecules cause the enlargement of blood vessels in a concentrated place, resulting in a high blood flow. This enlargement can bring on more features of inflammation, such as fever and swelling in the inflammation area. Overall, inflammation can protect against bacterial invasion. But it can also promote pathological inflammation, which can lead to the development of disorders such as abnormal blood clotting and sepsis (36–38).

Metabolic disorder and severe infectious increase the risk of pyroptosis in ECs, and promote several diseases, such as atherosclerosis, and sepsis-related acute lung injury, respectively (39, 40). In metabolic disorders or infectious conditions, caspase1 can promote lipids or inflammasome to aggregate and induce inflammatory factor release in ECs. In the canonical pathway, NLRP3 was stimulated by intracellular signaling molecule and assembled with pro-caspase1 and ASC, resulting in activated caspase1. Activated caspase1 stimulates pyroptosis, a caspase1-dependent inflammatory cell death described by a broken cellular membrane and released inflammatory mediators. Recently studies showed that gasdermin D (GSDMD) was cleaved by the inflammatory caspases1, 4, and 5 in humans and then separated GSDMD N-terminal (GSDMD-N)), which forms the transmembrane pore and functions as the executor of pyroptosis (19, 41, 42).

## 4 Endothelial dysfunction and pyroptosis in pathological

### 4.1 CVD

Cardiovascular disease (CVD) is a major public health problem and the reason for death globally. Atherosclerosis is a lipid metabolism syndrome that leads to the development of heart damage (such as myocardial infarction) and stroke by creating lipid-rich plaques that obstruct blood vessels inducing blood flow restriction and increasing the potential for plaque disruption (43). Once the plaques rupture and block the arterial lumen, tissues or organs supplied by the artery will show the parent’s organ irreversible injury and death. ECs protect cardiovascular homeostasis in normal conditions, even though lipid-rich plaques restrict blood flow. But in the terminal stage of the disease, endothelial dysfunction contributes to developing vulnerable plaques and impairs vascular homeostasis. As the main reason for the progression of atherosclerosis, pyroptosis may play a crucial role in endothelial dysfunction. Pyroptosis promotes inflammation, plaque disruption, and angiemphraxis by the release of inflammatory mediators such as interleukin 1β (IL1β) and interleukin 18 (IL18), and induces cell death, which hence develops CVD events (44).

**Figure 3:**
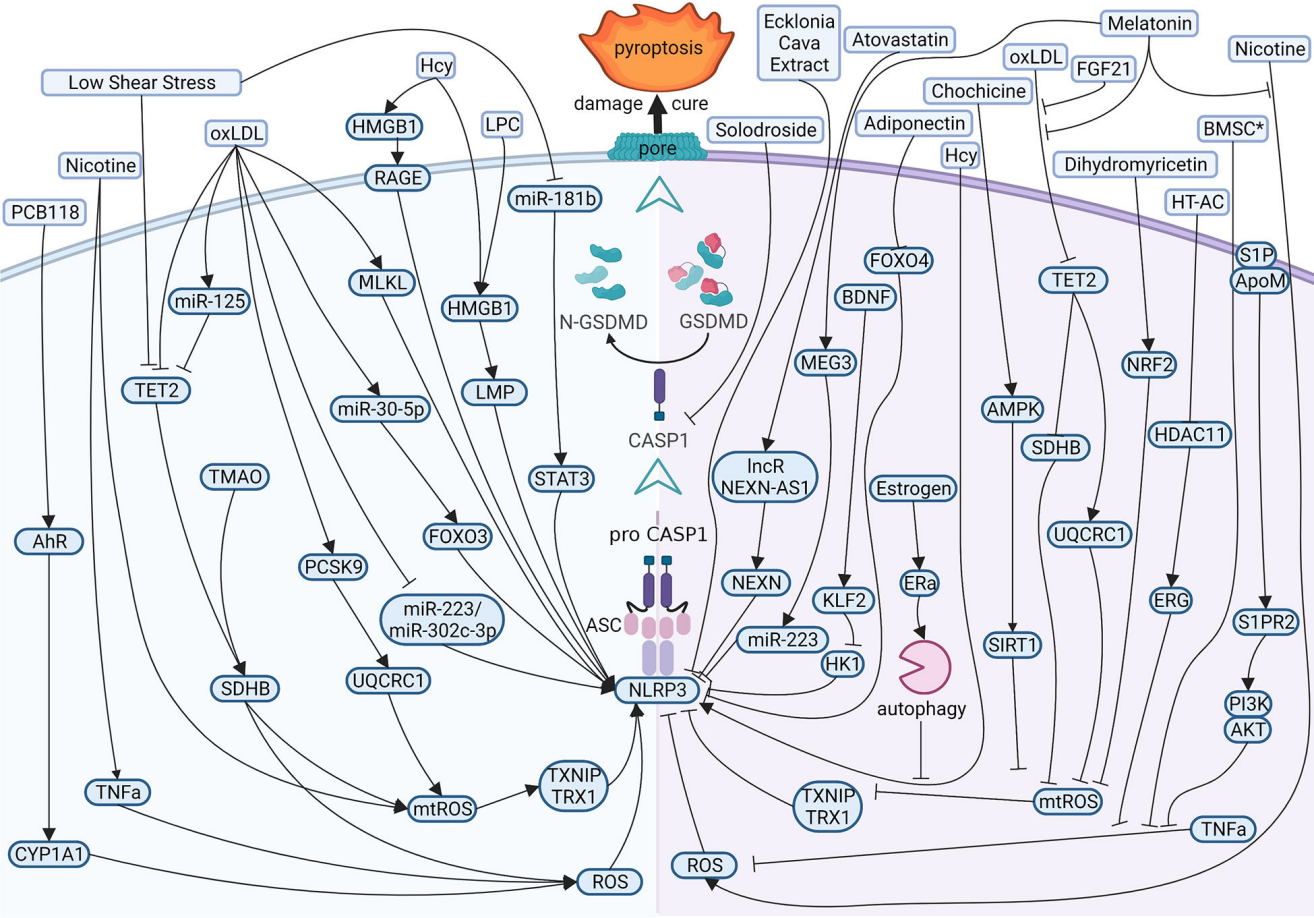
Diagram of the multi‐pathway activation mechanism of pyroptotic endothelial dysfunction in atherosclerosis (left), and chemical-treated (or active protein treatment) heal pyroptotic endothelial dysfunction in atherosclerosis (right). BioRender.com created the sketch.

Present evidence shows that the reasons for atherogenesis, such as cigarette smoking, and metabolic syndrome, are significant contributors to endothelial dysfunction (**Figure 3** left). Nicotine is a main avertible risk factor for atherosclerosis and CVDs. It promotes atherosclerosis in vulnerable areas, including the aorta, coronary arteries, carotid and cerebral arteries, and the large arteries in the peripheral circulation (45). In ECs, nicotine promotes the production of reactive oxygen species (ROS), which activates NOD-like receptor thermal protein domain associated protein 3 (NLRP3) inflammasome, leading to the activation of caspase1 (46). A study displayed that nicotine aggravated atherosclerosis in ApoE−/− mice fed with a high-fat diet (HFD), indicated by the more sizeable plaques and more lipids measured by oil red O. In normal diet mice, nicotine also promotes atherosclerotic plaques size and lipid aggregation; even so, the effect of nicotine was not more effective in HFD-fed mice. In addition, nicotine promotes endothelial damage and dysfunction by inducing pyroptotic macrophages (47).

Just as mentioned in the context, investigators have examined the effects of cholesterol on CVDs. In vessels, lipids accumulate under endothelium and impaired ECs directly. While endothelial damage, low-density lipoprotein (LDL) is remodeled to oxidized LDL (oxLDL) and adheres to intimal vessel forming interlayers (48, 49). OxLDL contributes to synthesizing adhesion molecules and releasing inflammatory mediators in impaired ECs (50, 51). OxLDL induces caspase1 activation by ROS, and NLRP3 levels rise in ECs (52, 53), and mixed lineage kinase domain-like (MLKL) aggravates oxLDL-induced pyroptosis by NLRP3-mediated inflammasome in human umbilical vein endothelial cells (HUVECs) (54). OxLDL also impaired mitochondrial structure and function, producing excess ROS in ECs. Proprotein convertase subtilisin/kexin type 9 (PCSK9)and trimethylamine N-oxide (TMAO) inhibited the function of mitochondrial by downregulating ubiquinol-cytochrome c reductase core protein 1 (UQCRC1) (55) and upregulating succinate dehydrogenase complex subunit B (SDHB) (56) expression, respectively, leading to ROS elevation and promotes the pyroptosis of ECs and the subsequent release of proinflammation cytokines. MicroRNA is also involved in pyroptotic endothelial dysfunction. miR-125a-5p, overexpression in oxLDL treatment ECs, directly targeted tet methylcytosine dioxygenase 2 (TET2) 3’-UTR and decreased protein expression, resulting in abnormal mitochondrial DNA (mtDNA) methylation levels and mitochondrial dysfunction promotes the production of ROS, which stimulated nuclear factor-κB (NF-κB) and subsequent induced NLRP3 inflammation and activated caspase1 (57).

There are several risk factors promoting pyroptosis in ECs and contributing to atherogenesis. Hyperhomocysteinemia (HHcy) induced high mobility group box 1 (HMGB1) overexpression because of produced NLRP3 inflammasome and caspase1 across the membrane, thereby cleaved GSDMD and induced pyroptosis (58, 59). Low shear stress decreased the levels of TET2, which recruited less histone deacetylase 2 (HDAC2) to increase the levels of SDHB. The high level of SDHB leads to mitochondria dysfunction and the production of ROS, which stimulates ECs pyroptosis (60); low shear stress also inhibits the levels of miR-181b-5p, resulting in an upregulation in signal transducer and activator of transcription 3 (STAT3) expression, which induced the synthesis of NLRP3 *via* histone acetylation in high levels. Moreover, NLRP3 induced caspase1 activation and activated caspase1 promote proinflammation factors pro-IL1β and pro-IL18 into mature. Eventually, GSDMD-N formation is cleaved by caspase1, leading to perforate cellular membranes, fragmented DNA, and release IL1β and IL18 from ECs, finally promoting pyroptosis ECs progression in atherosclerosis (61).

Many studies focus on therapeutic effects and potential drug targets (**Figure 3** right). Melatonin (N-acetyl-5-methoxytryptamine) is a neuroendocrine hormone synthesized in the pineal gland and many other organs (62, 63). In HFD-fed ApoE−/− mice, OxLDL stimulates pyroptotic cell death factors, including activation of MEG3 and inhibition of miR-223, resulting in NLRP3-ASC-procaspase1-assemble and activated caspase1, furthermore melatonin protected pyroptotic endothelium in ECs and improved atherosclerosis in mice models (64). Melatonin also protects against pyroptotic endothelial dysfunction by improving mitochondrial function and reducing ROS production (65). In addition, Melatonin alleviates nicotine-induced EC pyroptosis *via* suppressing ROS/NLRP3 pathway (66). Estrogen can promote autophagy by activation of estrogen receptor α, resulting in averts atherosclerosis by weakened EC pyroptosis (67). Fibroblast growth factor 21 (FGF21), an endocrine cytokine, protect mitochondrial structure and function by regulating the TET2-UQCRC1 pathway and results in reducing ROS production and inhibiting EC pyroptosis (68, 69). Chemical substances extracted in some plants were identified anti-pyroptosis effects in ECs, such as Chochicine, Dihydromyricetin, Ecklonia cava extract, hydroxytyrosol acetate by regulating AMPK, NRF2, NLRP3, HDAC11 respectively (70–74). miR-223 and miR-103 were reported to attenuate oxLDL/H2O2-induced pyroptotic cell death in ECs, demonstrating that non-coding RNA is a potential target in therapeutic atherosclerosis (75, 76). Interestingly, a report shows that bone marrow–derived mesenchymal stem cells conditioned medium attenuated the pyroptosis of vascular ECs induced by LPS and ATP, indicating a new therapy about biomedicine (77).

### 4.2 Infectious disease

**Figure 4:**
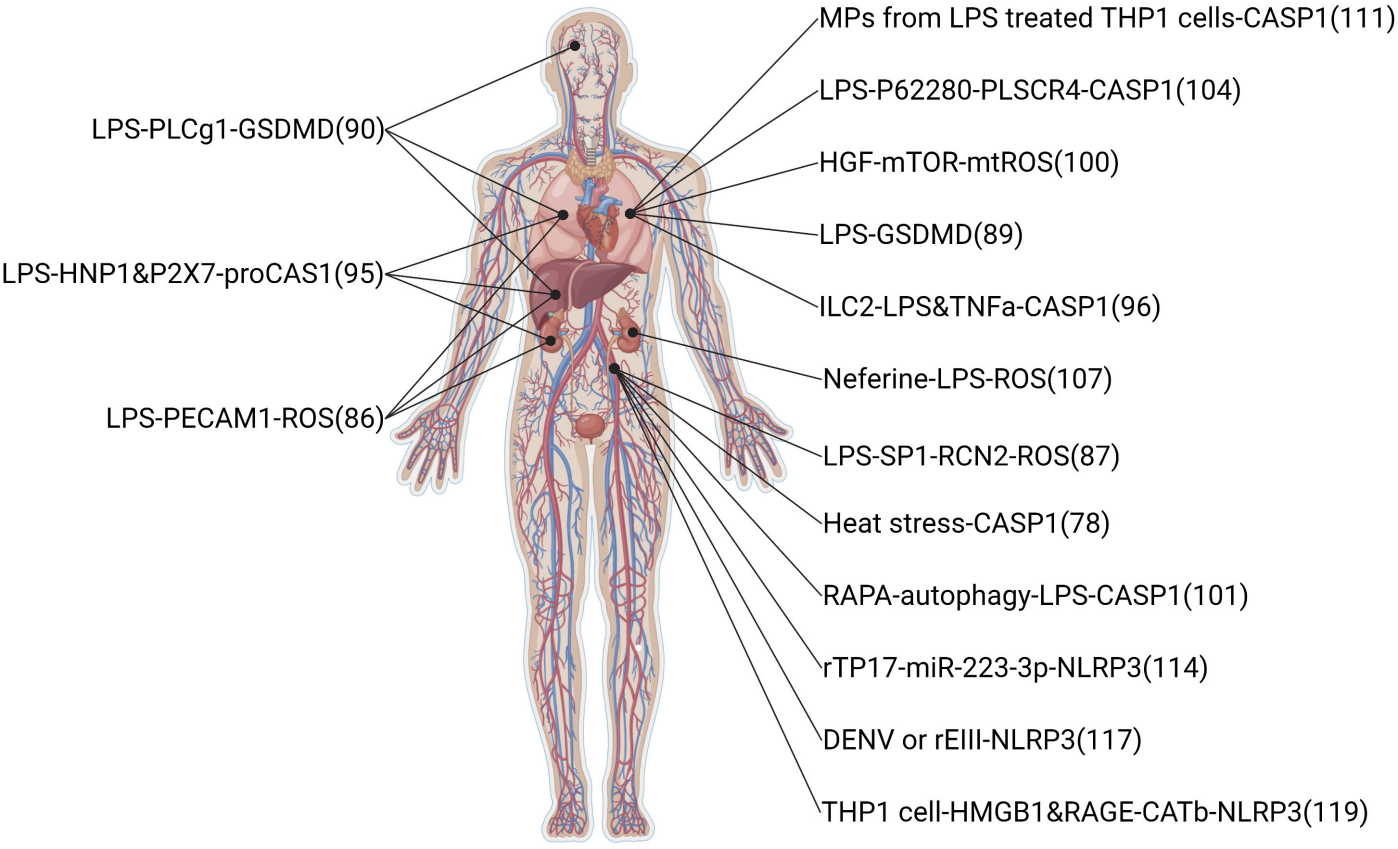
Related signaling pathway in infectious-induced pyroptotic endothelial dysfunction in multiple organs. The highest point indicates brain endothelium, the two second-highest points indicate lung endothelium, the middle point indicates liver endothelium, the two second-lowest points indicate kidney endothelium, and the lowest point indicates vessel endothelium. The original material was created by FIGdraw (https://www.figdraw.com/) and the sketch was made by BioRender.com.

The infectious disease also can induce ECs pyroptosis. Sepsis, critical organ damage induced by a metabolic disorder response to infections, is the primary means of ECs pyroptosis (78). The clinical feature of sepsis is volatile, relying on the preliminary area of infection, the pathogenic microbe, the type of organ damage, the body conditions of the patient, and the initial treatment (40). Symptoms of infectious and organ damage are complex and may have gone unnoticed, so the international consensus guidelines display a long list of sepsis warning signs. In this review, the summary pathway diagram that pyroptotic ECs induced multiple organ injury in infectious patients was created and shown in **Figure 4**.

ECs, which make up 50% of lung cells, are a part of the respiratory circulatory system and supply venous blood to the pulmonary parenchyma to carry out oxygen exchange (79). Pulmonary endothelium is tolerated stretching while breathing and always stays in the external environment, which increase the risk of being invaded by microbe from the air or peripheric pathogens. So, lung ECs are vulnerable to inflammatory damage in numerous clinical conditions, including sepsis, chronic obstructive pulmonary disease, Hemorrhagic shock, acute respiratory distress syndrome, and others (80–82). Several research focuses on the influence of pyroptosis on ECs in lung injury.

The lipopolysaccharide (LPS) inducing pyroptosis is the leading cause of dysfunction effects on the endothelium. LPS is the main component of the outer membrane of Gram-negative bacteria. It is sensed by the plasma-membrane-located toll like receptor 4 (TLR4) co-receptor MD2 complex in conjunction with co-receptor CD14, which specifically recognizes the lipid A structure of LPS and induces GSDMD-dependent pathway of pyroptosis by acting caspase11, caspase4, caspase5 (83, 84).

LPS promotes acute pulmonary vascular ECs injury in experimental animals and vitro (85, 86). LPS could inhibit endothelial nitric oxide synthase (eNOS) phosphorylation in ECs, and this phenomenon was diminished by reticulocalbin 2 (RCN2) silencing; overexpression of RCN2 in LPS-treated HUVECs could inhibit eNOS phosphorylation and induce HUVECs pyroptosis, NAC abolished this effect (87). Interestingly, a paper showed a new mechanism for LPS that induces the GSDMD pathway involving mitochondria (88). The mitochondrial membrane was combined with the GSDMD-N fragment induced by LPS, resulting in severe mitochondrial damage. Mitochondrial damage reduced mitochondrial membrane potential and promoted mtDNA release into endothelial cytoplasm, which was identified by cyclic GMP-AMP synthase (cGAS) and promoted reproduction of cyclic GMP-AMP (cGAMP) to stimulating inflammatory stimulator of interferon genes (STING) pathway. There is a new report about an auxiliary function of the perforating GSDMD in mtDNA in the cytoplasm and activating DNA-sensing cGAS/STING pathway, finally inducing EC pyroptosis and vessel disorder (88). cGAS/STING pathway could inhibit the process of dephosphorylation and nuclear translocation of the transcription factor Yes1 associated transcriptional regulator (YAP1) and induce cyclin D-mediated cell cycle arrest. In addition, the deletion of cGAS in mice brought back endothelial vitality in the damaged lung. Furthermore, GSDMD-dependent pyroptosis induced multiple organ injuries in sepsis mice, such as lung, kidney, and liver (89). Liu et al. have reported a paper suggesting that PhospholipaseCγ1-calcium promotes GSDMD-N translocation to the plasma membrane and increases LPS-induced EC pyroptosis and multi-organ damage, such as liver, lung and brain (90). A sturdy showed compared with H1N1 and control groups, more expression levels of caspase1 in endothelial tissue of the COVID-19 group, and this phenomenon might indicate that pyroptosis is happening in lung microvascular ECs of the COVID-19 patients (91).

Leukocytes participated in sepsis-induced pyroptosis endothelium dysfunction widely. Human neutrophil peptides 1-3(HNPs), the most generous neutrophil granule proteins, are abundantly expressed in neutrophils. The difference between HNPs is only an N-terminal amino acid sequence: the N-terminal amino acid is alanine in HNP1 and aspartic in HNP3. This amino acid is absent in HNP2, which is thought to be a proteolytic product of HNP1 and HNP3 (92, 93). A study showed that the overexpression of HNP1-3 (DEFA1/DEFA3) remarkably damaged the clinical characteristics of sepsis, making patients in China displayed a high gene copy number of DEFA1/DEFA3 means more easily affected by severe sepsis (94). In another paper, mice with high HCN of DEFA1/DEFA3 genes displayed graver pulmonary, hepatic, and renal injury and a dangerous consequence during infectious, suggesting a genotype-restricted function in phenotype development (95). In this study, the protein levels of vascular cell adhesion molecule 1 (VCAM1) and intercellular adhesion molecule 1 (ICAM1) in the lung tissue of DEFA1/DEFA3-HCN mice were significantly higher than those of DEFA1/DEFA3-LCN mice and WT mice 24 hours after the onset of disease septicemia. The figures showed that in mice bearing DEFA1/DEFA3 HCN, the activation state of vascular ECs in vital organs was more pronounced after the onset of sepsis. Furthermore, HNP1 activates caspase1 inflammasome by interaction with P2X7 and induces pyroptosis cell death in ECs. Group 2 innate lymphoid cells (ILC2) can protect lung ECs from pyroptosis in sepsis (96). ILC2, one of three subtypes of innate lymphoid cells (ILC1, ILC2, and ILC3), was detected in the lungs as a significant ILC population. ILC2 cells in the lungs and peritoneal cavity following CLP-induced sepsis showed IL33/suppression of tumorigenicity 2 (ST2), signaling overexpression and expansion of ILC2 in the lungs and provides ILC2-derived IL9, which alleviates sepsis-induced EC pyroptosis through controlling caspase1 activation. Furthermore, monocyte promotes human pulmonary microvascular endothelial cell (HPMEC) pyroptosis in hypoxia/reperfusion(H/R) injuries. In H/R injuries, the NLRP3 inflammasome and IL1β expression are increased, caspase1 is activated in monocytes, and eventually, IL1β and IL1R compomer of HPMECs induces pyroptosis through IL1R/NF-κB/NLRP3 signaling pathway (97).

Several potential drugs have been reported to protect against organ injury in sepsis *via* a pyroptosis-dependent pathway. Hepatocyte growth factor (HGF) is a pleiotropic cytokine involved in various cellular and biological processes, including attenuating cellular damage and reducing inflammation. Previous studies have demonstrated LPS-induced organ damage and elevated plasma HGF levels in rodents with systemic inflammatory response syndrome and early sepsis (98, 99). Recently, a paper showed that HGF improves the pyroptotic state of ECs by protecting mitochondrial physiology from releasing mitochondrial damage-related molecules and activating the mechanistic target of rapamycin kinase (mTOR) signaling. Intravenous injection of recombinant HGF into mice can alleviate mice’s lung endothelial pyroptosis caused by sepsis of various microorganisms and improve lung endothelial injury and acute lung injury (100). HGF will be a promising adjuvant therapy strategy for treating sepsis and acute lung injury. Furthermore, Rapamycin, a specific MTOR inhibitor, inhibited pyroptosis and protected ECs from excessive inflammation in the septic response (101). Phospholipid supersucrases (PLSCRs) translocate membranes in a Ca^2+^-dependent manner and perform nonspecific, bidirectional, and phospholipid-independent transduction in lipid bilayers, increasing their exposure to cell membranes (102, 103). A group of single-pass plasma membrane proteins that mediate layer migration. When HPMECs were stimulated with LPS, PLSCR4 expression, inflammatory cytokines IL1β, and IL18 levels increased EC permeability. While the PLSCR4 was silenced, human pulmonary microvascular endothelial cell (HPMEC) pyroptosis was remarkably risen, indicating the protective function of PLSCR4 in ECs (104). Neferine has various biological and pharmacological properties such as antitumor, antiinflammatory, antioxidative, antifibrosis, and antiarrhythmic (105, 106). Neferine is an alkaloid ingredient from the lotus seed embryo of Nelumbo nucifera. A paper by Tang et al. showed that neferine could inhibit LPS-ATP-induced oxidative stress and NLRP3 inflammasome signaling, increasing SOD production and improving EC viability (107).

Microparticles (MPs), a single membrane structure produced by apoptotic cells, have been detected in the site of disturbed blood flow in some pathological states, such as sepsis (108–110). Mitra showed that p30 GSDMD was found in MPs of septic patients (111). Furthermore, authors indicated that GSDMD was modified in MPs combined with activated caspase1 and released by LPS-stimulated Tohoku Hospital Pediatrics 1 cells, and these MPs with GSDMD and caspase1 promoted HPMEC pyroptosis. They demonstrated that GSDMD microencapsulation in MPs combined with caspase1 could be necessary for monocytes released MPs to vascular cells, resulting in caspase1-mediated pyroptotic HPMEC death.

Several clinical infectious conditions also can induce endothelium pyroptosis. Syphilis is a kind of multi-stage and chronic disease; the primary reason is infection by treponema pallidum subsp pallidum (T pallidum), which can influence various organs and has a high morbidity rate. The infections of syphilis are increasing rapidly worldwide (112, 113). A paper showed that miR-223-3p was remarkably decreased in syphilis patients compared with control groups, the levels of NLRP3 and caspase1 were increased in syphilis patients, and miR-223-3p inhibited T pallidum-induced caspase1 activation, IL1β production, and Lactate dehydrogenase (LDH) release in HUVECs, the mechanism is miR-223-3p targets NLRP3 directly (114). This paper highlighting demonstrated that miR-223-3p could become a drug target for treating infectious syphilis. Dengue virus (DENV) infection is a kind of the fastest growing mosquito-borne infections (115, 116). In a present study, Lien et al. found that the virion-associated envelope protein domain III (EIII) overexpression increases endothelial ROS production, induces tumor necrosis factor a (TNFa) and IL1β release, and promotes caspase1 activation, EC pyroptosis, and NLRP3 inflammasome inhibitor treatment significantly attenuates rEIII-induced ECs injury and significantly decreased bleeding in a dual-targeted rEIII autoantibody model (117). Kawasaki disease (KD), an acute vasculitis syndrome, is the primary reason for acquired heart disease in pediatric populations of developed countries (118). In a study by Jia et al., activation of pyroptosis is induced by HMGB1, resulting in increased levels of receptor for advanced glycation end-products (RAGE) and cathepsin B, which bring out NLRP3-caspase1 mediated inflammation-induced pyroptosis in ECs (119).

### 4.3 Diabetes mellitus

Diabetes is a global health problem and microvascular dysfunction is a major complication, leading to a series of diseases, such as retinopathy, nephropathy, neuropathy, and atherosclerotic diseases. The harmful effect of high glucose (HG) intrinsically is well-established on the endothelium. ECs of large vessels and the microvasculature were damaged by HG and had differences in autoregulation of glucose uptake in different organs (120, 121). For example, when exposed to high concentrations of extracellular glucose, retinal microvascular ECs did not reduce glucose uptake, whereas brain and cardiac ECs did. HG-induced diabetic retinopathy (DR) pyroptosis has attracted a lot of attention at present.

DR is a common retinal microvascular complication and a major reason for blindness in adults (122). In HG conditions, human retinal microvascular endothelial cell (HRMEC) dysfunction is a multifactorial pathogenesis, responsible for pathogenesis that is closely related to cell migration and apoptosis when cells are exposed to advanced glycation end products (AGEs). It is regulated by various inflammatory and apoptotic factors (123, 124). A paper showed HRMECs were pyroptotic by HG treatment (125). HRMECs HG-treated showed lower cell viability, and higher Caspase1 activity, indicating HG can induce HRMECs pyroptosis. Furthermore, HG treatment decreased miR-590-3p levels and increased NADPH oxidase 4 (NOX4) and NLRP1 expression in HRMECs. NOX4 and NLRP1 are direct targets to miR-590-3p and are the main intracellular regulator of the pyroptotic process, highlighting the significance of miR-590-3p in pyroptosis in DR. Another paper by Yang et al. showed AGEs induces ECs pyroptosis by active GSDMD and cleaved caspase1 in HRMECs (126). Moreover, authors indicate H3 relaxin, a multipotent peptide hormone of the insulin superfamily, remarkably inhibited migration, apoptosis, and pyroptosis in endothelium and relieved diabetic nephropathy *via* P2X7R-NLRP3 inflammation in HRMECs.

The corneal endothelium is the deepest monolayer of the cornea and keeps the stroma dehydrated by pumping fluid from the cornea into the anterior chamber. Intraocular pro-inflammatory cytokines can activate the activation of caspase1 and GSDMD (127). Corneal confocal microscopy showed that the density of corneal ECs was abnormally decreased and increased, suggesting that ECs are atypical in patients with diabetes (128). Corneal endothelium of donors indicated that the levels of NLRP3, caspase1, and IL1β were remarkably increased in the corneal endothelium of diabetic donors. Expression of lncRNA KCNQ1 opposite strand/antisense transcript 1 (KCNQ1OT1), a participant in multiple (physio) pathological processes of diabetic complications wildly, is upregulated, and miR-214, a downstream target of KCNQ1OT1, is downregulated in HG treatment corneal ECs. Caspase1 was reported to be a target gene of miR-214 and decreased expression by miR-214 directly, and result in providing a pyroptotic process in diabetic corneal endothelial dysfunction. Moreover, a paper showed metastasis-associated lung adenocarcinoma transcript 1 (MALAT1) stimulated pyroptosis by binding to miR-22 directly and inhibiting NLRP3 expression in EA.hy926 cells (129). A novel deep learning algorithm was used in corneal diseases to identify specific characteristics of the corneal sagittal plane, that may be helpful for diagnoses of corneal diseases (130).

Diabetic nephropathy (DN) also is a kind of diabetes complication. HG stimulated the caspase1-GSDMD-mediated pyroptotic pathway in glomerular endothelial cells (GECs) (131). Butyric acid, a short-chain fatty acid produced by the intestinal flora in the gut lumen, decreased the levels of GSDMD-N by suppressing caspase1-GSDMD pyroptotic process in HG conditions, thus providing GECs damage and inhibiting the releasing of pro-inflammatory factors (131). Butyric acid is a potential drug for endothelium dysfunction.

### 4.4 Stroke

Stroke is a kind of cerebrovascular disease that results from vascular rupture or blockage and the treatment of ischemic stroke according to the repair of blood flow in the ischemic zone. However, in some ischemic brain tissue, reperfusion may exacerbate injury or dysfunction and induce cerebral ischemia-reperfusion injury (CIRI). During CIRI, pro-inflammatory factors (for example oxygen free radicals); stimulate inflammatory cytokines, and increase the expression of adhesion molecules in leukocytes and vascular ECs, while neutrophils migrate and attach to microvascular ECs, resulting in neutrophils damage caused by aggression in ischemic tissue (132).

In experiments, oxygen-glucose deprivation (OGD) was used to mimic the condition of ischemia in cells. A paper showed bEnd.3 cells, a kind of mouse brain microvascular ECs, encourage the level of GSDMD-N at the membrane of bEnd.3 cells and produce pyroptosis-associated proteins under OGD condition, suggesting that the process of pyroptosis and inflammasome in brain microvascular ECs is happening in ischemic stroke (133). Furthermore, Mediresinol (MDN) activates PGC1α, promotes the interaction of PGC1α and PPARα in brain microvascular endothelial cells (BMECs), increases the expression of GOT1 and PAH, and ischemia-induced phenylalanine improves accumulation, thereby reducing mitochondrial ROS (mtROS). There are few studies on its pharmacological effects, and only the effect against Candida albicans infection has been reported (134). MDN is a potential drug for the treatment of blood-brain barrier (BBB) disruption and ischemic brain injury by inhibiting the pyroptosis of BMECs.

A paper used the OGD model or TNFα treated (mimic inflammation in reperfusion conditions) to show OGD-induced occludin degeneration and the lack of occludin could encourage BMECs death in both apoptotic and pyroptotic process during reoxygenation or TNFα treatment in cells (135). In this paper, OGD/reoxygenation (OGD/R) and TNFα activated pyroptosis in bEnd.3 cells and cleaved caspase1 and GSDMD-N expression significantly rose in OGD/R (or TNFα) treatment cells compared with normal cells, indicating the pyroptotic process of OGD (ischemia) and TNFα treatment EC in the stage of reoxygenation (reperfusion). Furthermore, overexpression of occludin inhibits both OGD/R and TNFα treated bEnd.3 cell pyroptosis indicates a potential occludin target in BBB disturbance in ischemic stroke.

Post-stroke cognitive impairment (PSCI) is a kind of long-term injury. A study used middle cerebral artery occlusion (MCAO)/1, 3, 7, 28 days reperfusion model to mimic PSCI (136). In this paper, hippocampus and cortex 1, 3, or 7 days after 45 min MCAO/reperfusion was measured by immunofluorescence staining and absent in melanoma 2 (AIM2), caspase1 and GSDMD were remarkably increased in the PSCI group, indicating pyroptosis happened. Furthermore, the immunofluorescence of AIM2 in PSCI mice was primarily co-localized with CD31 (EC marker) rather than NeuN (neuronal marker) and GFAP (astrocyte marker), indicating AIM2 production was generated in EC by PSCI pathogenesis. Moreover, AIM2 also mediated traumatic brain injury (TBI)-induced BMVECs pyroptosis (137).

### 4.5 Others

Hemorrhagic shock (HS) also could induce ECs pyroptosis in the lung. A study by Yang et al. showed the relationship between HS and ECs pyroptosis. They found that cold-inducible RNA-binding protein (CIRP), a protein of the cold shock protein family, promotes vascular damage and leads to pulmonary injury (138); Releasing of CIRP activates ECs pyroptosis by regulating adhesion molecules to promote intrusion of polymorphonuclear leukocytes and generate proinflammatory cytokines and ROS in hemorrhagic or septic shock conditions. CIRP also increased the level of NLRP3 inflammasome and promoted caspase1-mediated EC pyroptosis. Yang also reports another finding about the mechanism of EC pyroptosis induced by HS in the same year. In this paper, researchers listed two factors that induced caspase1 activation in ECs: HS stimulated the production of HMGB1 which assembled inflammasome and activated caspase1 *via* the RAGE pathway and induced the endocytosis of HMGB1 in ECs; otherwise, caspase1 was activated by LPS through TLR4-NLRP3 signaling pathway in ECs (139). Significantly, this study highlights the activated caspase1 in pyroptotic ECs in HS conditions. Lung transplantation is considered the only effective treatment for end-stage lung disease and there are numerous risk factors that can stimulate ischemia/reperfusion in the lung transplant (140). A study showed monocyte promote HPMEC pyroptosis in hypoxia/reoxygenation (H/R) conditions, the mechanism is both NLRP3 inflammasome and caspase1 are activated in monocytes, which stimulate IL1β secretion and bind to IL1R, leading to HPMECs pyroptosis through IL1R/NF-κB/NLRP3 signaling pathway (97).

## 5 Summary and perspectives

Many conditions can induce endothelium pyroptosis, and endothelium pyroptosis is a leading cause of organ injury, such as atherosclerosis, acute lung injury, diabetic retinopathy, and so on. The endothelium is not made up of just one fundamental EC but rather a large group of EC subtypes dissimilar in phenotype, function, and location. There is much less information about the mechanism by which this heterogeneity drives EC metabolism or how it is steered by EC metabolism in physiologically, and how different types of ECs respond to pathological conditions through the metabolic pathways according to their biosynthetic requirements. It is important for precision medicine.

Pyroptosis is an important potential target in diseases. Several compounds are pyroptotic inhibitors and being developed for pyroptosis-related diseases (25). In this review, we collected the potential targets of those compounds and pathogenic target of endothelial dysfunction by database. The shared regulatory networks of potential targets were recreated by Venny2.1 bioinformation tool and used in recreated PPI networks and KEGG and GO enrichment analyses. GO biological process suggested those compounds may have a role of inflammatory response in endothelial dysfunction (**Figure 2**). PPI networks and KEGG enrichment analysis showed those shared regulatory interactions belong to atherosclerosis, infectious and diabetes related pathway (**Figures 1**, **2**). Overall, the compounds, such as Z-VAD-FMK, have great therapeutic potential for pyroptotic endothelial dysfunction in diseases.

In CVDs, atherosclerosis is the main disease in pyroptotic endothelial dysfunction. Clinically, patients are asked to quit smoking, exercise and maintain their weight, blood pressure and blood lipids through a controlled diet, and adherence to this advice is help for lower cardiovascular mortality (141, 142). OxLDL is a major method to induce pyroptosis in ECs. OxLDL is a kind of oxidation of natural LDL and the most critical factor in atherosclerosis. OxLDL can influence multiple proteins (or non-encoding RNAs) to regulate the NLRP3-caspase1 pathway and induce EC pyroptosis. Pyroptosis attack ECs according to ROS (or mtROS)-NLRP3-caspase1 pathway mostly. Therefore, most drug targets (such as melatonin, dihydromyricetin, etc.) are chosen by inhibited ROS (or mtROS) to reverse endothelial damage.

Sepsis, as a systemic inflammatory response to infection, is the leading cause of pyroptotic endothelial dysfunctions in infection. Mechanisms of organ failure and death in patients with sepsis are little known, and autopsy studies do not show widespread necrosis (143). Therefore, endothelial homeostasis may be key in revealing the mechanisms of sepsis-induced organ injury. LPS, a kind of endotoxin released from Gram-negative bacteria into blood, is a major factor to induce pyroptotic endothelial dysfunction in infections. LPS-induced pyroptotic endothelial dysfunction damages multiple organs, such as the lung, brain, liver, and kidney. Furthermore, the lung is the most vulnerable to LPS-induced organ injury, the reason may be pulmonary ECs are the most cell in the lung and initial exposure to LPS in blood. In clinical, supportive care (including rapid recognition of sepsis and delivery of effective antibiotics, resuscitation with fluid therapy in early septic shock, lung protective ventilation, more judicious use of fluid therapy once shock has resolved, better guidelines for blood product transfusion, and enhanced methods to reduce secondary nosocomial infections) should be given more attention to ensure organs conditions in septic patients (144).

Vasculitis is a group of inflammatory autoimmune diseases induced by genetics, infection, and abnormalities of the innate and acquired immune systems. Autoantibodies, especially antineutrophil cytoplasmic antibody (ANCA) and anti-endothelial cell antibody (AECA), play an important role in the pathogenesis of vasculitis. In clinical, ANCA and AECA is used as a marker for vasculitis measured by indirect immunofluorescence and ELISA. However, there is a notable leak of empirical research focusing specifically on the relationship between pyroptosis and ANCA, exploring those relationship may contribute to revealing the pathogenesis of vasculitis.

Endothelial dysfunction is a major complication occurring in diabetes and induces organs of patients with diabetes injury, such as DR, DN, etc. Clinically, diabetic patients with a disease duration of more than 10 years often have a combination of retinopathy, which is one of the main causes of blindness. HG conditions and AGEs promote pyroptosis in retinal and corneal endothelial dysfunctions *via* an NLRP3-caspase1-GSDMD pathway and result in blindness. HG also promotes GEC pyroptosis *via* a caspase1-GSDMD pathway, and this effect is inhibited by butyric acid. Another complication of diabetes is cardiac dysfunction, which is defined as a microvascular disease and contribution to heart failure, cardiac shock, and sudden death in diabetic patients. It is still unclear how diabetes influences cardiac dysfunction. Endothelial dysfunction may be a key risk factor. However, there is no study about pyroptotic endothelial dysfunction in diabetic cardiomyopathy, and much uncertainty still exists about the relationship between those. Revealing the role of pyroptotic ECs in diabetic cardiomyopathy may contribute to ascertaining the mechanism of diabetic cardiomyopathy.

Endothelial barriers comprise BBB at the inner coating and endothelial homeostasis is important for brain health. During cerebral ischemia, oxidative stress and immune-inflammatory response promote endothelial dysfunction. In clinical, the treatment of ischemic stroke depends on the restoration of blood flow in the ischemic area, so reperfusion injury is also an important mode of injury in stroke patients. The present studies reported that OGD, a method to mimic the condition of ischemia in cells, could induce brain endothelial cell pyroptosis *via* mtROS or caspase1-GSDMD pathway. *In vivo*, MCAO/reperfusion was used to mimic long-term brain injury and indicated that brain ECs could secrete AIM2 and promote the caspase1-GSDMD pathway in the PSCI brain.

We have reviewed recent work on pyroptotic ECs in multiple diseases and discussed the lack in present studies. We have also analyzed several compounds, which are reported to test the effect in pyroptosis-associated diseases, using network pharmacology and the analysis of the GO and KEGG showing those compounds have tremendous potential for regulated pyroptosis-related endothelial dysfunction in diseases. This review hopes to provide new insight into how classic and emerging risk factors promote pyroptotic endothelium and how to develop a therapeutic strategy for organ injury treatment through the regulation of EC pyroptosis.

## Author contributions

JJ contributions to the design and writing of this manuscript. HL and BY reviewed the review. YL designed the figure. All authors contributed to the article and approved the submitted version.

## Glossary

**Table:**

AGEs	Advanced glycation end products
AIM2	Absent in melanoma 2
BMEC	Brain microvascular endothelial cell
cGAMP	Cyclic GMP–AMP
cGAS	Cyclic GMP-AMP synthase
CIRI	Cerebral ischemia-reperfusion injury
CIRP	Cold-inducible RNA-binding protein
CVD	Cardiovascular disease
DENV	Dengue virus
DN	Diabetic nephropathy
DR	Diabetic retinopathy
EC	Endothelial cell
EIII	Virion-associated envelope protein domain III
eNOS	Endothelia nitric oxide synthase
FGF21	Fibroblast growth factor 21
GECs	Glomerular endothelial cells
GO	Gene ontology
GSDMD	Gasdermin D
GSDMD-N	N-terminal GSDMD
H/R	Hypoxia/reoxygenation
HDAC2	Histone deacetylase 2
HFD	High fat diet
HG	High glucose
HGF	Hepatocyte growth factor
HHcy	Hyperhomocysteinemia
HMGB1	High mobility group box 1
HNPs	Human neutrophil peptides
HPMEC	Human pulmonary microvascular endothelial cell
HS	Hemorrhagic shock
HUVEC	Human umbilical vein endothelial cell
ICAM1	Intercellular adhesion molecule 1
ILC2	Group 2 innate lymphoid cells
KD	Kawasaki disease
KEGG	Kyoto Encyclopedia of Genes and Genomes
LDL	Low-density lipoprotein
LPS	Lipopolysaccharide
MCAO	Middle cerebral artery occlusion
MDN	Mediresinol
MLKL	Mixed lineage kinase domain-like
MPs	Microparticles
mtDNA	Mitochondrial DNA
mTOR	Mechanistic target of rapamycin kinase
mtROS	Mitochondrial ROS
NF-κB	Nuclear factor-κB
NLRP3	NOD-like receptor thermal protein domain associated protein 3
OGD	Oxygen glucose deprivation
OGD/R	OGD/reoxygenation
oxLDL	Oxidized LDL
PCD	Programmed cell death
PCSK9	Proprotein convertase subtilisin/kexin type 9
PLSCRs	Phospholipid scramblases
PSCI	Post-stroke cognitive impairment
RCD	Regulated cell death
RCN2	Reticulocalbin 2
RMEC	Retinal microvascular endothelial cell
ROS	Reactive oxygen species
STAT3	Signal transducer and activator of transcription 3
STING	Stimulator of interferon genes
T pallidum	Treponema pallidum subsp pallidum
TBI	Traumatic brain injury
TET2	Targeted tet methylcytosine dioxygenase 2
TLR4	Toll like receptor 4
TMAO	Trimethylamine N-oxide
UQCRC1	Ubiquinol-cytochrome c reductase core protein 1
VCAM1	Vascular cell adhesion molecule 1
YAP1	Yes1 associated transcriptional regulator
